# The pathway of cell dismantling during programmed cell death in lace plant (*Aponogeton madagascariensis*) leaves

**DOI:** 10.1186/1471-2229-12-115

**Published:** 2012-07-25

**Authors:** Jaime Wertman, Christina EN Lord, Adrian N Dauphinee, Arunika HLAN Gunawardena

**Affiliations:** 1 Department of Biology, Dalhousie University, 1355, Oxford Street, Halifax, Nova Scotia B3H 4R2, Canada

**Keywords:** *Aponogeton madagascariensis*, Autophagy, Lace plant, Live-cell imaging, Programmed cell death (PCD)

## Abstract

**Background:**

Developmentally regulated programmed cell death (PCD) is the controlled death of cells that occurs throughout the life cycle of both plants and animals. The lace plant (*Aponogeton madagascariensis*) forms perforations between longitudinal and transverse veins in spaces known as areoles, via developmental PCD; cell death begins in the center of these areoles and develops towards the margin, creating a gradient of PCD. This gradient was examined using both long- and short-term live cell imaging, in addition to histochemical staining, in order to establish the order of cellular events that occur during PCD.

**Results:**

The first visible change observed was the reduction in anthocyanin pigmentation, followed by initial chloroplast changes and the bundling of actin microfilaments. At this stage, an increased number of transvacuolar strands (TVS) was evident. Perhaps concurrently with this, increased numbers of vesicles, small mitochondrial aggregates, and perinuclear accumulation of both chloroplasts and mitochondria were observed. The invagination of the tonoplast membrane and the presence of vesicles, both containing organelle materials, suggested evidence for both micro- and macro-autophagy, respectively. Mitochondrial aggregates, as well as individual chloroplasts were subsequently seen undergoing Brownian motion in the vacuole. Following these changes, fragmentation of nuclear DNA, breakdown of actin microfilaments and early cell wall changes were detected. The vacuole then swelled, causing nuclear displacement towards the plasma membrane (PM) and tonoplast rupture followed closely, indicating mega-autophagy. Subsequent to tonoplast rupture, cessation of Brownian motion occurred, as well as the loss of mitochondrial membrane potential (ΔΨ_m_), nuclear shrinkage and PM collapse. Timing from tonoplast rupture to PM collapse was approximately 20 minutes. The entire process from initial chlorophyll reduction to PM collapse took approximately 48 hours. Approximately six hours following PM collapse, cell wall disappearance began and was nearly complete within 24 hours.

**Conclusion:**

Results showed that a consistent sequence of events occurred during the remodelling of lace plant leaves, which provides an excellent system to study developmental PCD *in vivo*. These findings can be used to compare and contrast with other developmental PCD examples in plants.

## Background

### Programmed cell death

Programmed cell death (PCD) occurs in both plants and animals, and is a highly regulated process that happens either as a part of normal development or in response to environmental influences [1,2]. Examples of environmentally induced PCD include, but are not limited to: heat shock [3] and the hypersensitive response (HR) [4-7]. Examples of developmentally regulated PCD include, but are not limited to: deletion of the embryonic suspensor [8-11], tracheary element (TE) differentiation [12], leaf senescence [13], and leaf morphogenesis [1,14-19].

The classification of plant PCD into categories based on morphological characteristics has been challenging in the past [2,7,20]. In 2000 Fukuda [21] defined three different forms of plant PCD 1. Apoptotic-like 2. Cell death during senescence and 3. PCD in which the vacuole plays a central role. In 2005 van Doorn and Woltering [20] compared plant PCD with three previously established morphological categories of metazoan cell death 1. Apoptosis 2. Autophagy and 3. Non-lysosomal PCD; these authors found that no plant examples conformed to the apoptotic type [20]. In 2008 Reape and McCabe [22] reverted to the use of Fukudas’ (2000) term, apoptotic-like cell death, referring to examples from both induced and developmentally regulated PCD. Recently, plant PCD has been classified into two main categories based on morphological characteristics: necrotic and vacuolar cell death [23]. However, these authors agree with van Doorn et al 2011 [23], that biochemical and molecular data are needed to classify the categories of plant PCD more accurately.

Autophagy is known to play a major role in the regulation of death in animal cells, but the extent to which autophagy is involved in plant PCD has yet to be concretized [20,24-26]. Autophagy has been divided into three forms within plants: micro-, macro-, and mega-autophagy [20,27,28]. Micro-autophagy is the uptake of organelles or other cellular contents by a lytic compartment, usually the vacuole in plant cells [20,28]. Macro-autophagy involves the creation of a double membraned structure containing cytosolic contents, called an autophagosome; this organelle will then fuse with a compartment, such as the vacuole, containing lytic enzymes [20,27,28]. Lastly, mega-autophagy involves the permeabilization or rupture of the tonoplast and the release of hydrolytic enzymes into the cell, causing total degradation [20,28].

Processes such as leaf senescence, TE differentiation, and the deletion of the embryonic suspensor have emerged as valuable examples used to study plant cell death [13,29,30]. However, to date very little research has been conducted *in vivo* to understand the order of organelle changes, which occur during developmentally regulated PCD *in planta*. Therefore, this study focused on establishing the sequence of cellular changes during plant PCD, to begin understanding cause and effect relationships between plant organelles.

### The lace plant as a novel model organism to study PCD *in vivo*

**Figure 1:**
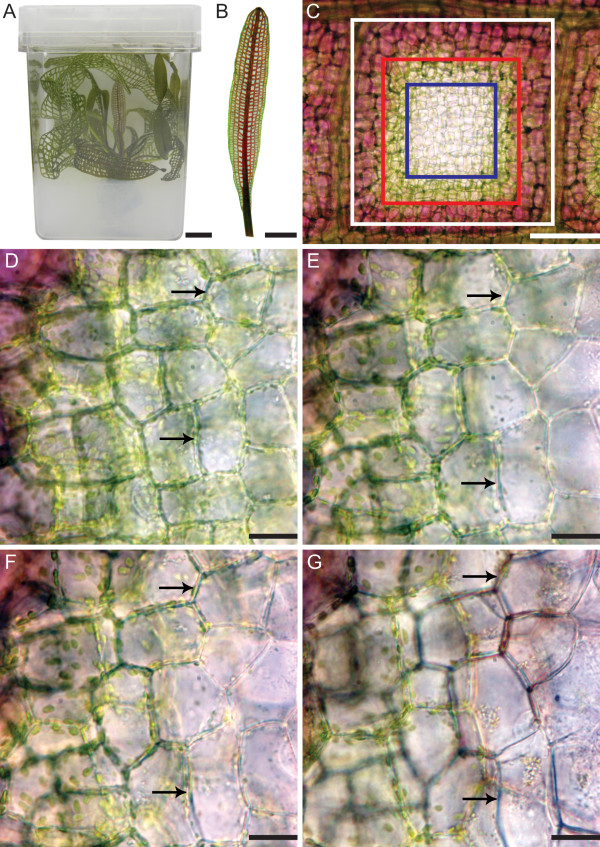
**The Lace Plant, or *Aponogeton madagascariensis. *** (**A**) Experimental lace plant in a magenta box containing liquid and solid MS medium. (**B**) Stage 2, or ‘window’ stage leaf. Note the pink colouration, due to the pigment anthocyanin in the plant cell vacuole. (**C**) Subdivision of a single areole of a window stage leaf. Surrounding the areole is vascular tissue, or the veins of the leaf (outside the white square). The first delineation are cells that will not undergo PCD, or the NPCD cells, and are used as control cells (between the white and the red rectangles). These cells are markedly pink due to the pigment anthocyanin. The next grouping of cells are those in the earliest stage of PCD, or the EPCD stage cells (between the red and the blue rectangles). These cells are normally green, marking the presence of chlorophyll. Finally, the cells in the latest stage of PCD, or LPCD stage cells, are in the centre of the areole (inside the blue rectangles). These cells are usually transparent, indicating that pigments have been degraded. (**D**-**G**) Progression of window development within a section of an areole captured during long-term live cell imaging approximately at times 0, 12, 36, and 60 hours, respectively. Arrows indicate cells that were initially seen with chlorophyll pigmentation (**D**), reduced pigmentation (**E** and **F**) and collapsed PM (**G**). Microscope settings remained constant throughout observation. Scale bars: A-B = 1 cm, C = 250 μm, D-F = 30 μm, G = 40 μm.

The aquatic lace plant is a submerged monocot endemic to Madagascar [31,32], that acquires its name from its unique perforated leaf morphology. In lace plant leaves, PCD begins in the center of areas known as areoles, between transverse and longitudinal veins and continues outwards, stopping four to five cells from the vasculature [14,16,18]. This system lends predictability to both the time and location of cell death, and in combination with its transparent and thin leaf, makes the plant an ideal specimen for live cell imaging [16,18,19]. As well, a technique has been developed to culture the lace plant in sterile conditions, providing ample experimental material without contamination (Figure 1A) [16]. Collectively, these qualities make the lace plant an excellent system to study developmentally regulated plant PCD *in vivo*[1,33] .

The process of perforation formation in lace plant leaves has been divided into five stages, as outlined in Gunawardena et al (2004) [14]. The present study will focus on stage 2, or window stage leaves, where PCD is actively occurring (Figure 1B). This window stage leaf is then examined at the level of a single areole (Figure 1C). The window stage areole is subdivided into three groups of cells along a gradient of PCD, based on morphological characteristics [19]. These groups include: non-PCD or control cells (NPCD; Figure 1C, between white and red lines), early-PCD cells (EPCD; Figure 1C, between red and blue lines) and late-PCD cells (LPCD; Figure 1C, inside blue rectangle). NPCD stage cells possess pink colouration, indicating the presence of the pigment anthocyanin. EPCD cells display chlorophyll pigmentation but no longer display large amounts of anthocyanin, indicating that this is one of the earliest changes in lace plant PCD [14,18]. The length of time between anthocyanin disappearance and chlorophyll reduction is still under investigation. Note that the leaves used in this study have previously lost most visible anthocyanin as this is a precise way to ensure that the area of interest will perforate. In the lace plant, various cellular morphological characteristics have been examined throughout the process of cell death [18,19], however the detailed characterization of the order of events occurring throughout the entire process has not yet been investigated. The aim of this paper is to use the novel lace plant system to delineate the possible order of organelle changes that occur throughout developmental PCD using both *in vivo* long- and short-term live cell imaging. This study will also employ experiments using fixed tissue samples in order to supplement this data. As a subset of this goal, this paper will provide visual evidence for autophagy during developmental PCD in the lace plant.

## Results

### Window formation

The gradient of cell death seen simultaneously in one areole of a window stage leaf was used to classify cells into groups (NPCD, EPCD and LPCD). Thus, each cell that underwent PCD during perforation formation transitioned from EPCD to LPCD (See Materials and Methods section for more details). Therefore, long-term live cell imaging was used to focus on individual cells (Arrows, Figure 1D-G; Additional file 1) initially containing chlorophyll pigmentation in EPCD (Figure 1D) until the collapse of the plasma membrane in LPCD (Figure 1G). The time between the reduction in visible chlorophyll in EPCD (Figure 1E) to PM collapse in LPCD (Figure 1G) in a typical individual cell was determined to be approximately 48 hours.

### Changes in F-actin, chloroplasts, mitochondria and nuclei

**Figure 2:**
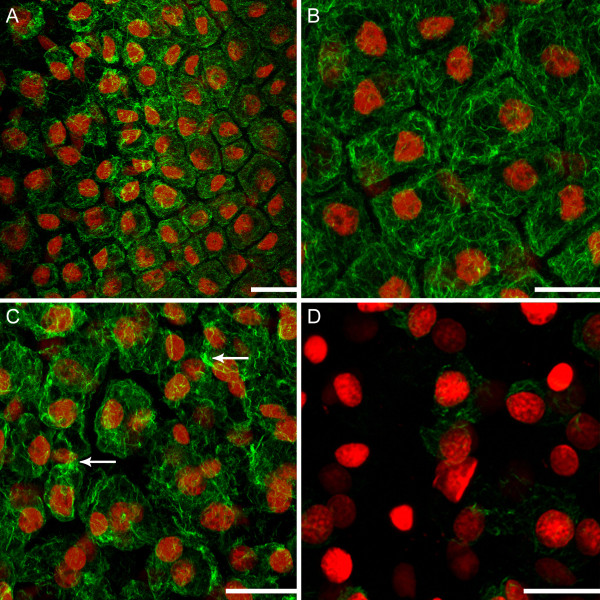
**Alterations in cytoskeletal dynamics within a single areole of a window stage leaf.** Each image represents a maximum projection of approximately 50 z-stack images. (**A**) Half of a single areole of a window stage leaf, displaying the gradient of non-PCD (NPCD), early-PCD (EPCD) and late-PCD (LPCD). Actin microfilaments are stained green with Alexa Fluor 488 Phalloidin, while nuclei are stained red with propidium iodide. Note the variation in cytoskeletal dynamics across this gradient. (**B**) NPCD cells displaying a thin layer of actin microfilaments underlying the PM. (**C**) EPCD cells displaying actin microfilament bundling. Note the bundles of actin found in several cells within the field of view (arrow). (**D**) LPCD cells displaying actin microfilament breakdown. Note the loss of actin staining within these cells and the discontinuous nature of the filaments that are still present. Scale bars: A-D = 100 μm.

Following anthocyanin loss, evidence depicted an initial reduction in chlorophyll content, as well as a reduction in the size and number of chloroplasts (Figure 1D-G). The actin cytoskeleton also underwent changes at this time, as demonstrated by Alexa Fluor 488 Phalloidin staining (Figure 2A). Control, or NPCD cells, displayed thin groups of actin filaments that appeared to underlay each PM (Figure 2B). Cells in the early stages of PCD displayed the first visible changes in actin arrangement; notably, the filaments re-organized into thicker cables (Figure 2C). There was also an increased number of visible transvacuolar strands (TVS) in EPCD stage cells (Additional file 2). The arrangement of the actin looked increasingly haphazard until LPCD, at which point there was less visible overall cytoskeleton stained (Figure 2D).

**Figure 3:**
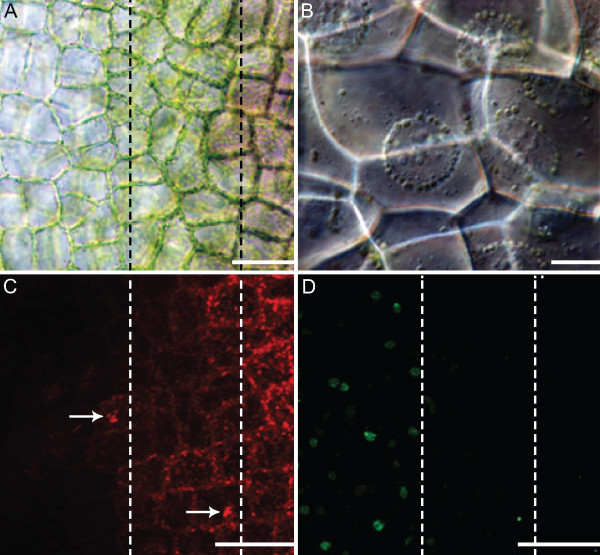
**Organelle dynamics within a single areole of a stage 2 or window stage lace plant leaf.** (**A**) Half of a single areole of a window stage leaf. Note that the image is sectioned into three stages, the right most section being NPCD, the middle being EPCD and the left most being LPCD. (**B**) Perinuclear accumulation of chloroplasts. (**C**) Mitochondrial dynamics over the gradient of PCD, visualized via CMXRos. Note that this gradient corresponds with that seen in panel A. NPCD stage cells contain many individual mitochondria in the cytosol. EPCD stage cells contain mitochondrial aggregates (arrows), along with some individual mitochondria. LPCD stage cells depict either aggregates or mitochondria, or a lack of staining, indicating the loss of ΔΨ_m_ (mitochondrial membrane potential). (**D**) Gradient of TUNEL positivity, indicating the cleavage of DNA by endonucleases throughout PCD in the lace plant. Note that this gradient corresponds with that in panel A. NPCD and EPCD stage cells do not contain TUNEL positive nuclei, indicating intact nuclear DNA. LPCD stage cells do contain TUNEL positive nuclei, signifying DNA cleavage by endonucleases. Scale bars: A= 100μm, B= 20μm, C-D= 120μm.

Figure 3A depicts a gradient of cell death that is seen in lace plant windows. The image is sectioned into three stages, the right most section being NPCD, the middle being EPCD and the left most being LPCD (Figure 3A). It was noted that, in EPCD stage cells, individual chloroplasts and/or small groups of mitochondria could be seen moving along TVS in a seemingly orderly fashion (Additional file 2). In EPCD stage cells, it was common to see chloroplasts in a ring-like formation surrounding the nucleus, shown in Figure 3B. The gradient of CMXRos staining seen in Figure 3C corresponds with the gradient delineated in Figure 3A. During EPCD there was also an increase in the associations between mitochondria, causing small groupings of mitochondria within the cytosol (Figure 3C). EPCD cells also contained aggregates of chloroplasts and mitochondria undergoing Brownian motion in the vacuole (Additional file 1). It should be noted that these aggregates became visibly larger as PCD advanced (Additional file 1).

The gradient of TUNEL positive nuclei that could be seen in a lace plant areole began at the border of EPCD to LPCD cells, indicating that it is one of the earlier cell death characteristics that occurred in LPCD (Figure 3D); note that the gradient division seen in Figure 3D corresponds to that seen in Figure 3A. Also at this time, organelle aggregates were still seen undergoing Brownian motion in the vacuole until tonoplast rupture initiated the cessation of movement during the late stages of cell death (Additional file 3). Later, mitochondria exhibited the loss of mitochondrial membrane potential (ΔΨm), as denoted by the lack of CMXRos staining (Figure 3C). Overall, the presence of TUNEL positive nuclei supports the notion that LPCD cells contain fragmented nDNA (Figure 3D).

### Evidence for autophagy and aggregate formation

**Figure 4:**
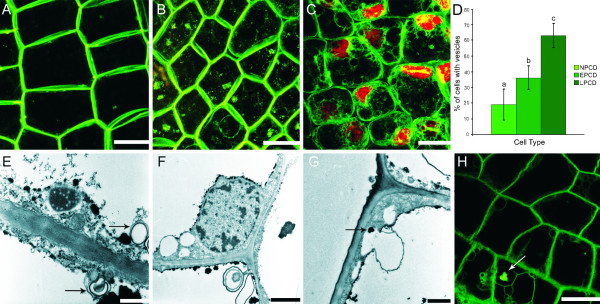
**Presence of autophagic vesicles throughout lace plant PCD.** (**A**) Non-PCD (NPCD) stage cells co-stained with FM1-43 (green), a dye that localizes to phospholipid bilayers, and PI (red), a dye that localizes to the nucleus. Staining of the PM and possibly the tonoplast, in addition to several small round organelles can be seen. The absence of PI staining indicated healthy nuclei. (**B**) EPCD stage cells co-stained with FM1-43 and PI. Note stained PMs, some tonoplast staining, in addition to several autophagic vesicles. Organelle aggregates were present and stained with FM1-43. Again recognize the absence of PI staining, indicating healthy nuclei. (**C**) LPCD stage cells co-stained with FM1-43 and PI. Note PM, tonoplast and organelle staining as well as the multiple, variable sized vesicles. Most nuclei in LPCD cells are stained with PI, indicating non-viable nuclei (**D**) Percentage of cells containing vesicles by stage. NPCD, EPCD and LPCD stages had 22, 38 and 66 percent of their cells containing vesicles, respectively. Bars with different letters differed significantly (P < 0.05). Error bars represent standard error of a minimum of 120 cells per category. (**E**) TEM micrograph of a late stage lace plant leaf. Double-membraned vesicular structures, some of which contain organelle material can be seen (marked by black arrow). (**F**) TEM micrograph of a late stage lace plant cell. Note multiple vesicles, including one double membraned vesicle containing organelle material. (**G**) TEM image of late stage PCD cell depicting invagination of the tonoplast, possibly indicating micro-autophagy. Electron dense material in the invagination that is possibly composed of organelles can be seen (marked by black arrow). (**H**) LPCD stage cell stained with FM1-43. Note the stained PM, organelles and vesicles. White arrow points to an aggregate that appears to be within a small vacuole. Scale bars: A = 50 μm, B-C = 100 μm, E = 0.4 μm, F = 1 μm, G = 0.8 μm, H = 100 μm.

Window stage leaves stained with FM1-43 displayed vivid staining of the tonoplast and plasma membranes (Figure 4A-C, H). Small amounts of this stain also localized to the outside of organelles such as mitochondria and chloroplasts, due to their surrounding phospholipid membranes. Vesicle-like objects were rarely present in NPCD stage cells (Figure 4A), and increased in number from the early stages (Figure 4B) to the later stages (Figure 4C) of PCD. To support the notion that vesicles were more commonly seen in LPCD cells than EPCD or NPCD cells, the percentage of cells that contained vesicles was quantified (Figure 4D). These results depicted that the percent of cells containing vesicles differed significantly between each group (P < 0.05), with 66% of LPCD stage cells, 38% of EPCD and 22% of NPCD cells containing vesicles.

In order to more closely examine these vesicles, lace plant cells in later stages of PCD were observed via TEM and similar membrane-bounded bodies were found (Figure 4E-G). Rarely, results displayed double-membraned bodies, sometimes containing organelle material (Figure 4E, F). Additionally, in several instances, vesicles, with or without contents, appeared to be fusing with the tonoplast membrane (Figure 4F). Further evidence of autophagic-like processes was seen using live-cell imaging; FM1-43 staining displayed vesiculation of LPCD lace plant cells that occasionally contained organelle material (Figure 4H).

**Figure 5:**
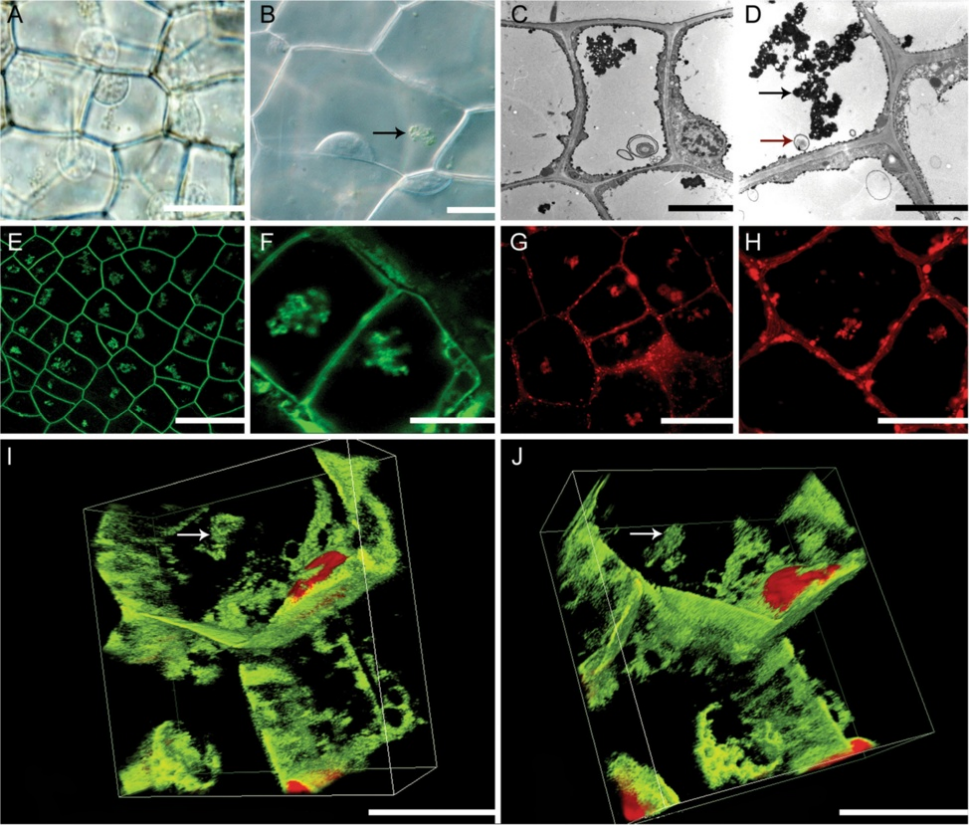
**Visualization of the organelle aggregate seen in EPCD and LPCD stage cells.** (**A**) DIC image depicting aggregates of mitochondria and chloroplasts in early-PCD (EPCD) stage cells. (**B**) Higher magnification DIC image from EPCD stage cells. Aggregate marked by black arrow (**C**) TEM image of EPCD stage cells depicting an aggregate of electron dense material inside the vacuole of a lace plant cell; note the membranous body in the vacuole. (**D**) Higher magnification TEM image of an EPCD stage cell, displaying aggregate of electron dense material in the vacuole, marked with a black arrow. Also note the organelle material was bound by a single membrane in the vacuole, marked with a red arrow. (**E**) Low magnification confocal micrograph displaying EPCD stage cells stained with FM1-43, sequestered to the phospholipid bilayer. Note the aggregates of membrane-bound organelles within each cell. (**F**) High magnification confocal micrograph depicting LPCD stage cells stained with FM1-43. Note the presence of the organelle aggregate and some vesiculation in the cells. (**G**) Confocal micrograph of EPCD stage cells stained with CMXRos, which stains mitochondria with intact ΔΨ_m_ (mitochondrial membrane potential). Note the aggregates are stained red, indicating the presence of mitochondria. (**H**) High magnification confocal micrograph of EPCD cells stained with CMXRos. Note that the aggregates are stained red, indicating the presence of viable mitochondria. (**I**) 3D volume view confocal image of LPCD stage cells co-stained with FM1-43 and propidium iodide (PI). Fifty successive z-stack images were compiled into a single 3D image using the deconvolution demonstration from Nikon Instruments. Note the presence of the aggregate within the vacuole of the cell, denoted with white arrow. Also note surrounding vesicles. (**J**) Secondary view of panel I, also depicting the aggregate, stained with FM1-43 within the vacuole of LPCD lace plant cells, denoted by white arrow. Scale bars: A = 100 μm, B = 10 μm, C = 50 μm, D = 25 μm E = 150 μm, F = 25 μm, G = 50 μm, H-J = 25 μm.

In this study, aggregates were observed with DIC optics, TEM imaging, FM1-43, CMXRos staining and via long and short-term live cell imaging (Figure 5; Additional files 1, 3 and 4). Aggregates appeared to be formed within EPCD cells, and were most prominent in LPCD stage cells. They were irregularly shaped and seemed to be composed of organelle materials. Live cell imaging suggested that these aggregates are chloroplasts or chlorophyll-containing objects due to their colour (Figure 5A, B; Additional file 1). Within individual cells, the size of the aggregate appeared to increase as PCD progressed. Individual or small groups of organelles undergoing Brownian motion independently of the aggregate were seen to attach to the aggregate and then move in unison with the cluster (Additional file 1). Also, it is important to note that Brownian motion was also seen in NPCD stage cells, albeit to a much lesser extent (data not shown). Using TEM imaging, it was apparent that the aggregates were mostly electron-dense (Figure 5C, D). Using FM1-43, it was confirmed that these aggregates consisted of organelles surrounded by phospholipid bilayers (Figure 5E, F). Using the mitochondria-specific stain CMXRos, it was also apparent that there were mitochondria present within the aggregates (Figure 5G, H; Additional file 4).

Through the use of 3D analysis it was revealed that the aggregates (indicated by the arrows) were in the vacuole in later stages of cell death (Figure 5I, J). These aggregates of mitochondria and chloroplasts were also infrequently observed within vesicles (Figure 4H, 5C, D). In addition, TEM imaging depicted organelle material in an invagination of the tonoplast (Figure 4E, G) and within the vacuole (Figure 5C, D). In contrast, in NPCD stage cells, these organelles were normally dispersed throughout the cytosol and less commonly seen in the vacuole (Additional file 1).

### Tonoplast rupture to cell wall degradation

**Figure 6:**
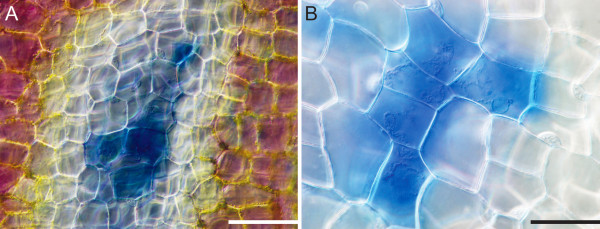
**Plasma membrane intactness, demonstrated by Evans Blue staining.** (**A**) Window stage leaf stained with Evans Blue. Note the gradient of PCD that exists, with the outermost cells being non-PCD (NPCD) stage cells and the centermost being late-PCD (LPCD) stage cells and the centermost being late-PCD stage cells (LPCD). Also note that only LPCD stage cells, with collapsed PMs, stain blue. (**B**) Higher magnification of a similar leaf as shown in panel A, again note that only cells with collapsed PMs possess blue staining. Scale Bars: A = 100 μm, B = 50 μm.

**Figure 7:**
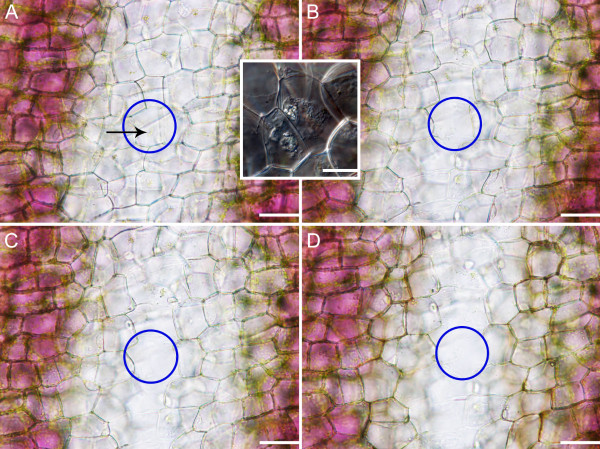
**Time course analysis of cell wall degradation.** The blue ellipse highlights a single cell for focus. (**A**) Time 0, has an intact PM. Also note the characteristic aggregate of mitochondria and chlorophyll containing material within the cell, indicated by the black arrow. Inset: Late-PCD (LPCD) stage cell with a collapsed PM and visibly intact cell wall. The collapse of the PM occurs between the stages shown in panels **A** and **B**. (**B**) Time 6 hours, recognize that within this 6 hour time span, the PM of the cell of interest has collapsed, and some cell wall degradation has began to occur. (**C**) Time 12 hours, note the cell of interest now has little visible cell wall content. (**D**) Time 24 hours, note the cell of interest now appears to have very little visible cell wall content and cellular wall degradation is nearly complete. Scale bars: A-D = 100 μm, Inset = 20 μm.

In LPCD, nuclei were observed to be displaced, visibly smaller and pushed against the PM (Additional files 1, 3 and 5). This was followed by rupture of the tonoplast and vacuolar collapse (Additional file 5). Following vacuolar collapse, the nucleus was liberated and then shrunk, the aggregate of organelles stopped moving (Additional file 3), and the PM collapsed (Additional files 3, 5 and 6); the process from tonoplast rupture to PM collapse took approximately 20 minutes (Additional file 5). Plasma membrane collapse is visualized in Additional file 6. At this stage, plasma membrane collapse was also made apparent by positive Evans Blue staining (Figure 6A, B). In late LPCD cells, it was apparent that mitochondria had lost their ΔΨm, as evident in Figure 3C. Initial cell wall changes were also observed early in LPCD via lightening of cell walls in the centermost cells (Figure 7A). Additionally, live cell imaging depicted visual evidence of cell wall disappearance occurred within 24 hours following PM collapse (Figure 7A-D). It is important to note that this experiment began with cells with intact PMs and followed them throughout cell wall degradation.

## Discussion

**Figure 8:**
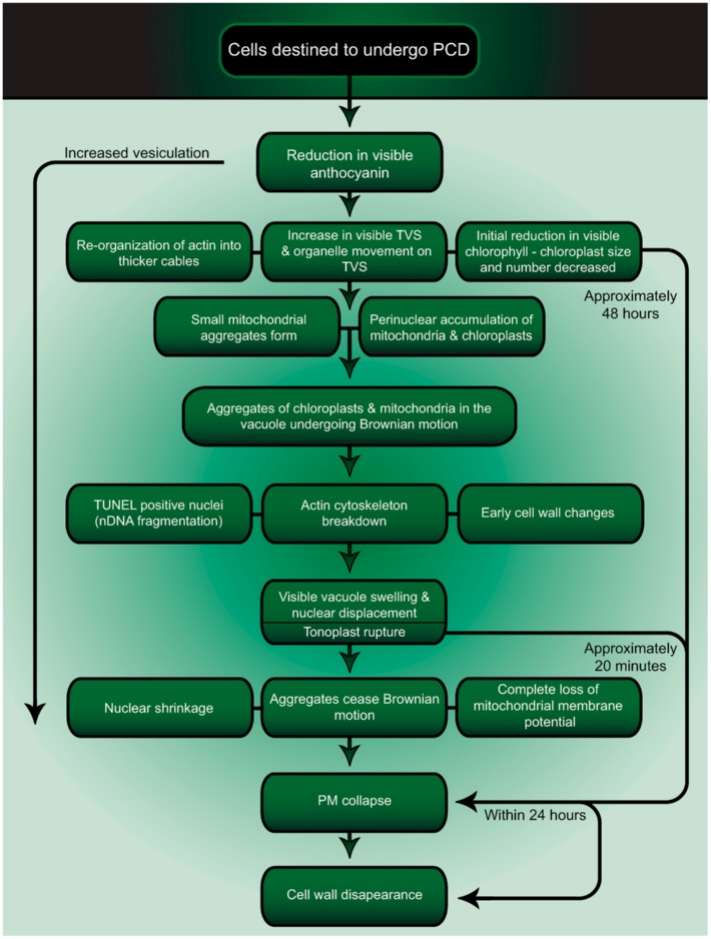
**Summary flowchart.** Proposed timeline for cellular changes during developmental PCD throughout lace plant leaf morphogenesis. Note that control, or NPCD stage cells, do not undergo PCD, but instead appear to undergo normal cellular processes throughout the development of the perforation.

The lace plant window provides a spatially and temporally predictable system within which to study developmental PCD *in vivo*. Similar studies have used mesophyll cells isolated from *Zinnia elegans* to study developmentally regulated PCD in the past; however, these studies are considered *in vitro* as differentiation into TEs was induced following cell isolation [5]. Therefore, these authors consider the live-cell imaging reported within this manuscript as a unique data set. Results presented here elucidate the sequence of cellular events occurring during developmentally regulated PCD in the lace plant using long- and short-term live cell imaging techniques (Summarized in Figure 8; Additional file 1). 

The first visible change noted in lace plant cells undergoing PCD is the reduction/disappearance of anthocyanin [14]. It is unclear whether this reduction is due to early changes in the pH of the vacuole following variations in tonoplast permeability, as seen in petal senescence [34], or due to the actual degradation of the pigment. More research is needed to determine the reason for these colour changes that occur early in the PCD process. This loss of pigment in the center of a perforation occurs very early in the PCD process, and is often already reduced when the leaf unfurls. Following this, the pigment chlorophyll becomes less abundant [14,18]. This is due to the decrease in chlorophyll within the chloroplasts and the significant reduction in both chloroplast size and number [18] that is seen in EPCD cells (Additional file 1). Lim et al (2007) [13] also reported that loss of chlorophyll is a characteristic of leaf senescence, where initial changes occur within the chloroplasts [13].

Perhaps concurrently with chlorophyll reduction, actin filament organization begins to change. The actin filaments of the cytoskeleton are known to play major roles in cell expansion, division and differentiation; however, less is known about their role in PCD [35,36]. At the border of NPCD-EPCD, filaments changed from thin, organized structures coating the periphery of each protoplast (Figure 2B), to thicker cables that are more haphazard in arrangement (Figure 2C); this was also seen in *Picea abies* embryos [37]. Also at this stage an increase in the number of TVS becomes apparent. This increase in TVS has been reported during developmentally regulated PCD in the lace plant and during induced cell death in both lace plant protoplasts and tobacco suspension cultures [3,18,38]. Previous work in the Gunawardena lab showed organelles moving along TVS [19]; additional evidence of this is provided in the present study (Additional file 2). The movement of organelles along these TVS may result in the perinuclear accumulation of organelles seen in EPCD stage lace plant cells (Figure 3B) [19].

It was determined via FM1-43 staining that vesicles begin increasing in prevalence in EPCD stage cells. The majority of observed vesicle structures were membrane-bound bodies that may or may not contain organelle material. It is probable that the vesicles seen via FM1-43 staining and confocal imaging are the same vesicles seen in the cytosol and joining with the tonoplast via TEM imaging (Figure 4E-G). The present study also noted several instances of multi-membraned structures that sometimes contained organelle material. Similar swirled cytoplasmic membranes were seen by Filonova et al (2000) [9] during PCD in embryogenesis in Norway spruce and were called ‘whorls’ [9]. Liu et al (2005) [8] also observed similar vesicle-containing membranous structures as seen in lace plant PCD, during the HR response in *Nicotiana* plants [8]. In addition, it has been determined that autophagy is necessary for developmental PCD in TE formation in *Arabidopsis*, which may provide further evidence for the role autophagy plays in plant PCD [26]. Overall, these membrane-bound structures represent evidence for autophagy during lace plant PCD.

As PCD progresses lace plant cells display aggregation of organelles. The early aggregation of mitochondria in cells undergoing PCD has been noted previously during induced cell death in *Arabidopsis*[39,40] and also in previous developmental PCD studies within the lace plant [3,19]. The various forms of microscopy used here depict chlorophyll-containing organelles, as well as mitochondria in these aggregates. The association among and between mitochondria and plastids has been observed previously, where mitochondria were often seen touching and embedded within chloroplasts of senescing wheat leaves [41].

Long-term live cell imaging provides evidence suggesting that the aggregates, characteristic of EPCD and LPCD stage cells, visibly increased in size, perhaps through the accumulation of individual organelles as PCD progresses (Additional file 1). Wright et al (2009) [18] previously noted aggregates in the vacuole of lace plant cells undergoing PCD that displayed distorted thylakoid membranes, thus indicating the presence of chloroplasts [18]; these aggregates appeared to be undergoing Brownian motion as seen in Additional files 1, 4, 3 and 6 of this manuscript. Through the use of confocal z-stack imaging and NIS-Elements Volume View Software, the present authors were able to determine that these aggregates were positioned in the vacuole during the later stages of PCD. Presumably, these organelles are in the vacuole to be degraded; however, it is as of yet unknown how and exactly when this movement into the vacuole occurs. The authors believe that small aggregates of mitochondria, and perhaps individual chloroplasts, are brought to the vacuole independently, potentially aided by TVS, following which aggregation would occur (Additional files 1, 3, 4 and 6). Alternatively, the aggregate could be assembled in the cytosol and then brought into the vacuole as a whole. Regardless, this phenomenon of organelles in the vacuole is likely indicative of some form of autophagy [20].

In LPCD stage cells, it is also discernible in Figure 3C and 3D that there are cells that have both CMXRos staining and TUNEL positive nuclei. In addition, Figure 3C and 3D also display cells, to the far left (late LPCD), that contain TUNEL positive nuclei without CMXRos staining. Therefore, it was deduced that DNA fragmentation occurs prior to the loss of mitochondrial ΔΨ_m_ indicated by a loss in CMXRos staining. Perhaps concurrently with the appearance of TUNEL positive nuclei, the actin cytoskeleton appeared to breakdown. Re-organization of actin into thick cables followed by its breakdown is a common feature of developmentally regulated plant PCD [36]. Some authors proposed cytoskeleton alterations as a trigger for the onset of PCD in *Arabidopsis* during the HR [42]. However current work in our lab suggests that cysteine-aspartate specific protease-like proteases (caspase-like proteases, CLPs) may act upon the actin cytoskeleton, suggesting the cytoskeleton may not be a initial trigger for PCD but instead a target of an upstream signal, although further research is required (data not shown). In addition to cytoskeletal changes in LPCD stage cells, the current study provides live cell imaging evidence of visible cell wall changes, as reported originally by Gunawardena et al (2007) [17] via TEM.

Following the appearance of TUNEL positive nuclei and the breakdown of actin, visible changes in tonoplast dynamics became apparent. It is unknown whether this marks the first change in tonoplast integrity, as it is also possible that an early change in selective permeability of the tonoplast could have caused the subsequent anthocyanin colour change. The rupture of the tonoplast is known to be involved in several examples of plant PCD including TE differentiation and aerenchyma formation [19,43-45]. The present study demonstrates, to the best of these authors’ knowledge, the first *in vivo* video recording of tonoplast rupture and subsequent visible cellular events (Additional file 5). The rarity of the above observations is explained by the quick succession in which these events occur. Additional file 5 depicts swelling of the vacuole resulting in the flattening of the nucleus against the PM, a characteristic also seen in TE differentiation [43]. In lace plant cells, this step is followed closely by tonoplast rupture, visible nuclear liberation, and subsequent cessation of organelle aggregate Brownian motion (Additional file 3), loss of mitochondrial ΔΨ_m_ (Figure 3C) and rapid nuclear shrinkage. Although it was not possible to view CMXRos stained cells and also see tonoplast rupture at the same time, the order for cessation of organelle aggregate movement and loss in mitochondrial ΔΨ_m_ was discerned from Lord et al 2011 [19]. This maintenance of mitochondrial integrity until late in lace plant PCD is reminiscent of leaf senescence and xylem differentiation [13,20,46,47]. A similar process is also seen in TE differentiation, where studies report liberation of the nucleus followed by complete nuclear degradation 20 min post tonoplast rupture due to nucleases being released from the vacuole [43]. However, the current study does not provide evidence of complete nuclear degradation, as intact condensed nuclei can still be seen following cell wall degradation [Figure 7; [14,17].

Tonoplast rupture is regarded as the decisive moment during plant PCD in which cytoplasmic streaming stops and the cell is considered dead [2,18,20]; more recently, tonoplast rupture has also been coined as the process of ‘mega-autophagy’ by van Doorn and Woltering (2005) [20]. Within this manuscript we have presented evidence supporting van Doorn’s latest classification of the lace plant into vacuolar type PCD, especially with regards to the presence of autophagy characteristics. However, it is still unclear whether mega-autophagy by van Doorns and Woltering’s (2005) [20] definition applies to the lace plant given that most organelles have been degraded prior to tonoplast rupture.

Following tonoplast rupture within the lace plant system, PM collapse is observed (Figure 7. inset; Additional files 5 and 6). Additional evidence of PM collapse is seen via positive Evans Blue staining, that is only able to enter cells with compromised PMs (Figure 6). In the lace plant, there is approximately 48 hours between initial chlorophyll reduction and PM collapse (Additional file 1; Figure 1D-G). Depending on the form of PCD, PM collapse may or may not be followed by cell wall degradation [4,9,17,43]. During TE differentiation, the cell wall is partially degraded and modified, leaving an empty tube [9]. Conversely, during leaf senescence in *Arabidopsis,* increasing leaf weight is seen up until approximately 30 days following sowing, suggesting no visible wall degradation [48]. In contrast to both of these systems, Gunawardena et al (2007) [17] showed changes in wall structure as early as in stage 2, or in window stage leaves during lace plant leaf morphogenesis via TEM. These results were complemented by results presented in this manuscript in which cell wall disappearance was shown to occur within 24 hours of PM collapse using live cell imaging (Figure 7A-D) [17]. The inset in Figure 7 displays an LPCD stage cell with a blebbed PM and intact cell wall, indicating PM collapse precedes visible cell wall degradation. However, the extent of cell wall degradation was examined only qualitatively here and thus represents a possible area for future research.

## Conclusions

This study aimed to employ the novel *in vivo* system of a single areole in a window stage lace plant leaf, to elucidate the sequence of organelle changes occurring throughout developmentally regulated PCD in plants. The first visible change observed was the reduction in visible anthocyanin, then initial reduction of chlorophyll pigmentation within chloroplasts along with changes in chloroplast size and number. Perhaps concurrently with chloroplast changes, actin filaments re-organized into bundles, and there were increased numbers of TVS and additional instances of organelles travelling along TVS. Mitochondria then tended to form small groupings in the cytosol, and cells often possessed rings of chloroplasts surrounding the nucleus. Live cell imaging revealed single-layered membranous structures while TEM imaging revealed both single and multi-layered membranous structures; each of which were seen to occasionally contain organelle material. In addition, images depicting invaginations of the tonoplast and double membrane-bound vesicles indicating a role for micro- and macro- autophagy in lace plant PCD were also presented. Later, the aggregate of mitochondria and chloroplasts could be seen within the vacuole undergoing Brownian motion, and this aggregate visibly increased in size as PCD progressed. This study shows, for the first time, an *in vivo* video during developmentally regulated PCD, of vacuole swelling and tonoplast collapse, resembling mega-autophagy. This paper provided evidence of these events leading to the cessation of organelle aggregate movement, loss of mitochondrial ΔΨ_m_, nuclear shrinkage and PM collapse. Total time from reduction in visible chlorophyll to PM collapse is approximately 48 hours. Cell wall disappearance followed the collapse of the PM within 24 hours. This study reports a consistent order of events that occur in lace plant cells undergoing PCD, in addition to providing visual evidence of autophagy.

## Methods

### Plant material and selection

Lace plants were propagated using the sterile tissue culture technique described in Gunawardena et al (2006) [16]. In brief, plants were grown in magenta boxes containing both solid and liquid Murashige and Skoog (MS) medium (Figure 1A). These lace plants were exposed to 12 h light/12 h dark cycles provided by daylight simulating fluorescent bulbs (Philips, Daylight Deluxe, F40T12/DX, Markham, Ontario) at approximately 125 μmol m^−2^ s^−1^ at 24°C. All chemicals were purchased from Sigma (St. Louis, MO, USA). All experiments were completed at least 5 times, unless otherwise stated. Additionally, all image plates were composed using Adobe Photoshop Elements version 6.0.

Experiments used window stage, or stage 2, lace plant leaves (Figure 1B). Areoles in which the pigments had already cleared from the centermost cells were chosen. This was done to ensure that the given areole would in fact perforate, as some never do. In addition, windows were chosen in which there was a clear delineation between NPCD, EPCD and LPCD stage cells. These stages, represented by the rectangles in Figure 1C, served as a gradient through which all stages of the PCD process could be observed simultaneously. Cells within four to five cells of the vasculature were denoted as non-PCD cells (NPCD; Figure 1C, between white and red lines). These cells were prominently pink in colour due to the pigment anthocyanin found in their vacuole, were not pre-disposed to undergo PCD, and were therefore used as control cells. Cells interior to the control cells were in the early stages of cell death and were regarded as early-PCD cells (EPCD; Figure 1C, between red and blue lines); anthocyanin in these cells had disappeared and they were green in colour due to the abundance of chlorophyll containing chloroplasts within them. In the center of the areole, the cells were in the latest stage of cell death, were generally cleared of all pigments, and were called late-PCD cells (LPCD; Figure 1C, inside blue rectangle). It is important to note, however, that LPCD stage cells were once EPCD stage cells and all EPCD stage cells will develop into LPCD stage cells. Although EPCD cells, which contain anthocyanin, are similar in appearance to NPCD cells, NPCD stage cells differ in that they do not undergo PCD during perforation formation. Therefore, NPCD cells remain static, whereas both the EPCD and LPCD actively undergo PCD.

### Light microscopy

All light microscopy observations were conducted using DIC optics on a Nikon Eclipse 90*i* compound microscope (Nikon Canada, Mississauga, ON, Canada). Images were captured using a digital camera (DXM 1200c). NIS-Elements AR Version 3.0 software was used for both imaging and analysis on this microscope. All slides were prepared as wet mounts.

### Confocal microscopy

Confocal laser scanning microscopy observations were conducted using a Nikon Eclipse T*i* microscope fitted with a digital camera (Nikon DS-Fi1). EZ-C1 3.80 imaging software was used for image acquisition and Ti Control was used for microscope control. Images were captured using DIC optics and corresponding fluorescent images were taken using either tetramethylrhodamine isothiocyanate (TRITC excitation 527–552 nm, emission 577–632 nm) or fluorescein isothiocyanate (FITC; excitation 460–500 nm, emission 510–560 nm) lasers. Maximum projection images were composed of approximately 30 to 50 successive z-stack images compiled using NIS-Elements AR version 3.0 software.

### Transmission electron microscopy

Window stage leaves were cut from lace plants and segments of approximately 2 mm × 2 mm were fixed in 2% (w/v) gluteraldehyde in 0.05 M sodium cacodylate buffer (pH 6.9) in a vacuum at 20 psi for 24 h. These segments were rinsed in buffer and post fixed for 4 h at room temperature in 2.5% aqueous osmium tetroxide and then dehydrated in a graded ethanol series. Leaf pieces were then placed in ethanol:Spurr resin mixtures and embedded in Spurr resin and polymerized for 9 h at 70°C. A Reichert-Jung ultra-microtome was used to prepare the gold sections that were then gathered on formvar coated grids and finally stained with lead citrate and uranyl acetate. These prepared segments were viewed using a Philips Technai 12 TEM (Philips Electron Optics, Eindhoven, Netherlands) operating at 80 kV. This microscope was fitted with a Kodak Megaview II camera (Rochester, New York, USA), with appropriate software (AnalySIS, Soft Imaging System, Münster, Germany).

### Long and short term live cell imaging

Videos were acquired on a compound light microscope using the audio video interleave (AVI) capture function in NIS-Elements AR software version 3.0. Long term live cell imaging (~72 h) experiments were carried out using window stage leaves where PCD was not visibly initiated, as denoted by central cells of the areoles still possessing green colour due to the presence of chloroplasts. The leaves were mounted between a custom slide and coverslip, submerged in 400 μL of distilled water and then sealed with petroleum jelly to prevent evaporation. Every six hours, the samples were rinsed three times with distilled water, re-mounted, and refocused to the appropriate cells.

Following the determination of the basic timeline of the cellular changes in PCD, it was necessary to obtain high-magnification videos for particular events. Videos were taken of carefully chosen window stage leaves, containing areoles in which the centermost cells were about to undergo membrane blebbing. The leaves were placed in between a slide and a coverslip and mounted as mentioned above. Samples were viewed for 1–6 h (short-term), with constant supervision to prevent changes in focal plane.

All videos were compressed using the Radius Cinepak Codec and kept at full length and size until subsequent analysis. Videos were shortened to the desired length using either QuickTime Pro (Version 10.0), IMovie (Version 9.0.2) or Adobe Premiere Pro CS5 (Version 5.0) software. Arrows and text were inserted into the videos using Adobe Premiere Pro CS5 (Version 5.0) or QuickTime Pro (Version 10.0). Videos captured throughout individual long-term live cell imaging experiments were trimmed, compiled and edited using Adobe Premiere Pro CS5 (Version 5.0).

### Staining

#### Alexa fluor 488 phalloidin

Window stage leaves were removed from plants and the midrib was excised. These segments were then fixed in 2% paraformaldehyde (BioShop Inc., Burlington, Ontario, Canada) solution containing actin-stabilizing buffer (ASB) for 3 h at 4°C. These segments were rinsed, cut into 5 mm^2^ and incubated overnight with 0.1 μM Alexa Fluor 488 phalloidin (Invitrogen Canada Inc., Burlington, Ontario, Canada) with 0.1% Triton X-100, all in ASB. After rinsing, the segments were counterstained with 0.5 mg/ml PI for approximately 5 min, mounted in Fluorogel (Electron Microscopy Sciences, Hatfield, Pennsylvania, USA) and viewed via confocal microscopy.

#### MitoTracker red CMXRos

CMXRos stain was dissolved initially in DMSO and further in dH_2_0 to a final concentration of 0.6 μM. Window stage leaves were removed from lace plants, the mid rib was removed and the leaf was cut into segments of approximately 5 mm^2^. These leaf sections were incubated in CMXRos at room temperature in the dark for 60 min. Following this, the leaves were rinsed 8 times in dH_2_0 and shaken at 100 rpm for 90 min. The leaf sections were then mounted in dH_2_0 and viewed via confocal microscopy.

#### Terminal deoxynucleotidyl transferase–mediated dUTP nick end labeling assay (TUNEL)

Window stage leaf segments were prepared as per the CMXRos staining protocol, fixed in FAA for 2 h, and rinsed 3 times in phosphate buffered saline (PBS). The TUNEL assay was completed according to the manufacturer’s instructions (Roche Diagnostics, Mannheim, Germany). Segments were counterstained with 3% (w/v) propidium iodide (PI) for 2 min. for co-localization of nuclei; PI fluorescence was excluded during figure preparation. Positive controls were competed with DNase 1 and negative controls were completed without the terminal deoxynucleotidyl transferase enzyme. All samples were then viewed with confocal microscopy.

#### FM1-43

Window stage leaf segments were prepared as per the CMXRos staining protocol and incubated in a 5 μg/ml FM1-43 solution dissolved in ice cold PBS. Leaf pieces were then incubated in a vacuum at 20 psi for 4 h. These leaf segments were not rinsed, but counterstained immediately with 3% (w/v) PI for 3 min and subsequently mounted in Fluorogel (Electron Microscopy Sciences, Hatfield, PA, USA). These segments were viewed via confocal microscopy within 1 h of staining.

To conduct the vesicle counting trials, window stage leaves were stained as above. Cells from each stage of PCD were captured with z-stack imaging and compiled into a maximum projection image, to simultaneously show all planes. The total number of whole cells seen in each image was counted. The number of cells in each image that contained at least one vesicle was counted. The number of cells containing vesicles was then divided by the total number of cells and multiplied by 100 in order to obtain a percentage of cells containing vesicles per stage. This value was averaged over at least 120 cells per stage. Data was analyzed using a general linear model of variance ANOVA and the average percentages were compared using the Tukey test at 95% confidence intervals (P < 0.05). Statistical analysis was carried out using Minitab 16 statistical software (Minitab Inc., State College, PA, USA, 1972).

Three-dimensional image analysis was performed on window stage leaves stained with FM1-43, as described above. Confocal microscopy was employed to capture 30–50 successive z-stack images of cells from an areole of a window stage leaf. Files from the EZ-C1 software were exported and opened with NIS-Elements AR (Version 3.0), containing the Deconvolution Demonstration from Nikon. Images were compiled and edited with the Volume View function and the Deconvolution Demonstration Package.

#### Evans blue

Window stage leaves were cut from lace plants and had their midribs removed. The leaf segments were then stained for one hour in a 0.5% (w/v) solution of Evans Blue dissolved in dH_2_0. Leaves were then washed in distilled water, mounted on a slide and viewed with the compound light microscope.

## Abbreviations

ΔΨ_m_: Mitochondrial membrane potential; CMXRos: MitoTracker Red CMXRos; EPCD: Early-Programmed cell death; FITC: Fluorescein isothiocyanate; LPCD: Late-Programmed cell death; NPCD: Non-Programmed cell death; PCD: Programmed Cell Death; PI: Propidium Iodide; PM: Plasma membrane; TE: Tracheary elements; TEM: Transmission Electron Microscopy; TRITC: Tetramethylrhodamine isothiocyanate; TUNEL: Terminal Deoxynucleotidyl Transferase dUTP Nick End Labeling; TVS: Transvacuolar strands.

## Competing interests

The authors declare that they have no competing interests.

## Author’s contributions

JW and CENL both contributed equally to manuscript preparation. JW carried out experiments including: selected short-term imaging, all FM1-43 staining and 3D analysis, selected CMXRos and all Evans blue staining, as well as TUNEL staining and imaging. CENL carried out experiments including: selected short-term imaging, cytoskeleton staining, TUNEL staining and imaging, selected CMXRos staining, statistical analysis, as well as light microscopy for cell wall degradation images. AND completed selected CMXRos staining, long-term live cell imaging, video editing and flow-chart compellation. JW drafted the first manuscript while JW, AND and CENL all contributed to final manuscript revisions. AHLANG, conceived the study, participated in its design and coordination, and helped in manuscript revisions as well as supervised all experimental work.

## Supplementary Material

Additional file 1 **Video clip compilation showing the development of a window over a period of 72 hours.** Note that this video focused initially on early-PCD (EPCD) stage cells. Chloroplast pigmentation, movement and size differ between cells in different stages of PCD. Small aggregates become larger as PCD advances and individual organelles are seen attaching to the aggregate. Transvacuolar strands (TVS) can be seen within cells and are most frequent in EPCD. Note the advancement of EPCD visibly denoted by a decrease in chloroplast size and number, resulting in a reduction in chlorophyll pigmentation. The two cells of interest show a loss of chloroplast pigmentation, individual organelles and aggregates undergoing Brownian motion, and near the final stages of PCD, nuclear displacement followed by PM collapse. Approximately 100X sped up. Click here for file

Additional file 2 **Transvacuolar strands (TVS) in EPCD stage lace plant cells.** Video displays chloroplasts and mitochondria (small, round, grey-coloured objects) within lace plant cells, in addition to the occurrences of visible TVS. Note the instances of organelle movement along TVS. Also note the perinuclear accumulation of some chloroplasts. Note the visible reduction in anthocyanin from the top right corner to the bottom left corner. Approximately 100X sped up. Click here for file

Additional file 3 **Aggregate cessation of Brownian motion.** This video shows a gradient of cell death: control cells on the left, early-PCD (EPCD) stage cells in the center and late-PCD (LPCD) stage cells to the right. Video depicts perinuclear accumulation of chloroplasts. Note the moving organelle aggregates at the beginning of the movie. Note visible vacuole swelling, causing displacement of the nucleus and subsequent tonoplast rupture. Following tonoplast rupture, the organelle aggregate stops Brownian motion; PM collapse quickly follows this process. Approximately 200X sped up. Click here for file

Additional file 4 **Mitochondrial aggregates stained with CMXRos.** Video displays that the aggregate of organelles contains CMXRos stained, mitochondria. This video demonstrates that the aggregate is moving within the cells vacuole via Brownian motion. Refer to Figure 5 for histological analysis of these aggregates. Approximately 10X sped up. Click here for file

Additional file 5 **LPCD stage cells displaying tonoplast rupture and subsequent events.** This video depicts vacuolar swelling, displacing the nucleus toward the PM. Following this, the tonoplast ruptures, allowing the nucleus to be temporarily liberated. The nucleus then condenses. Following this, the tonoplast shrinks and PM collapse follows closely. Approximately 200X sped up. Click here for file

Additional file 6 **Visualizing the process of PM collapse.** Note that the mesophyll cells’ PMs begin collapse prior to the epidermal cells. Also note the synchronicity of PM collapse. Approximately 200X sped up. Click here for file
